# Identification and Mitigation of Inhibitory Substances Contained in High-Salinity Crude Glycerol Generated from Biodiesel Production for Polyhydroxyalkanoate Synthesis by *Haloferax mediterranei*

**DOI:** 10.1021/acssuschemeng.5c05475

**Published:** 2025-09-22

**Authors:** Xueyao Zhang, Richard F. Helm, Emily L. McCoy, Fujunzhu Zhao, Mingxi Wang, Yebo Li, Stephanie Lansing, Haibo Huang, Zhiwu Wang

**Affiliations:** † Department of Biological System Engineering, 1757 Virginia Polytechnic Institute and State University, Blacksburg, Virginia 24061, United States; ‡ Department of Biochemistry, 1757 Virginia Polytechnic Institute and State University, Blacksburg, Virginia 24061, United States; § Department of Environmental Science and Technology, 1068 University of Maryland, College Park, Maryland 20742, United States; ∥ Department of Food Science & Technology, 1757 Virginia Polytechnic Institute and State University, Blacksburg, Virginia 24061, United States; ⊥ Quasar Energy Group, Wooster, Ohio 44691, United States

**Keywords:** glycerol sludge, long-chain fatty acids, polyhydroxyalkanoates, LC-MS, fermentation

## Abstract

High-salinity crude glycerol generated from biodiesel production poses significant challenges to microbial valorization due to inhibitory ingredients that severely limit microbial growth. This study identified and mitigated inhibitory substances contained in high-salinity glycerol sludge to enable its conversion to polyhydroxyalkanoates (PHAs) by the extreme halophilic archaeon *Haloferax mediterranei*. The long-chain fatty acids (LCFAs) were consistently identified as the primary inhibitors by liquid chromatography–mass spectrometry, Fourier transform infrared spectroscopy, and ultraviolet–visible spectroscopy. Acid precipitation at pH 2 efficiently removed these LCFAs, substantially reducing the required feedstock dilution from 23 to 3 times, improving PHA titer by 40%. Furthermore, this dilution reduction also increased the feedstock salinity utilization, achieving a 46% reduction in external salt supplementation for *H. mediterranei* growth. In contrast, overliming and arrested anaerobic digestion were confirmed to be ineffective in inhibitor removal. This study provides deep insights into inhibitor chemistry and presents acid precipitation as an effective pretreatment strategy for waste valorization of high-salinity crude glycerol.

## Graphical abstract

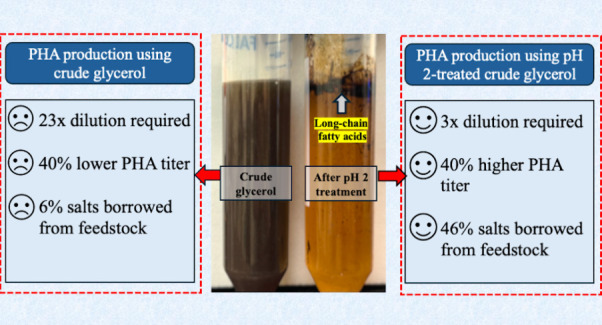

## 1 Introduction

Biodiesel, a renewable fuel derived primarily from plant oils and animal fats, has emerged globally as a promising alternative to fossil fuel.^1^ However, a critical challenge accompanying biodiesel expansion is the generation of waste glycerol (crude glycerol), a major industrial byproduct typically accounting for approximately 10 wt % of biodiesel produced.^2^ With global biodiesel production reaching 71.5 million tonnes in 2023, more than seven million tonnes of crude glycerol were generated, posing substantial logistical and economic challenges related to the disposal or further purification of this waste stream.^3^

Crude glycerol is formed as a byproduct of the transesterification reaction, in which triglycerides from fats and oils react with alcohols, typically methanol or ethanol, to produce fatty acid methyl or ethyl esters, known as biodiesel.^4^ Typically, crude glycerol streams contain significant impurities, including residual methanol, soap residues, free long-chain fatty acids (LCFAs), and elevated concentrations (50∼200 g/L) of sodium or potassium salts resulting from catalyst neutralization and undesirable side reactions such as saponification.^5–7^ These contaminants severely limit the direct use of crude glycerol and pose substantial challenges for conventional wastewater treatment systems. In particular, anaerobic digestion (AD) processes often reject crude glycerol or accept it only at substantially diluted levels to prevent operational issues such as microbial inhibition and reduced methane yields.^8,9^ Additionally, purification of crude glycerol requires costly and energy-intensive methods such as vacuum distillation or ion-exchange processes, significantly increasing operational costs and complicating waste management.^10^ Consequently, developing effective and economically viable approaches for the sustainable valorization of crude glycerol represents an urgent priority for enhancing both the environmental performance and economic sustainability in biodiesel refineries.

Recently, biological conversion of crude glycerol into valuable bioproducts, such as biodegradable polymers, has gained substantial attention.^11^ Polyhydroxyalkanoates (PHAs) represent a class of microbial bioplastics with widespread applications in packaging, medical materials, and agriculture due to their biodegradability, biocompatibility, and physicochemical versatility.^12,13^ Among microbial candidates, halophilic archaea have emerged as ideal organisms for utilizing crude glycerol due to their intrinsic adaptation to extreme hypersaline conditions and metabolic capacity for direct glycerol-to-PHA conversion.^14,15^ Specifically, the extremely halophilic archaeon *Haloferax mediterranei*, which thrives optimally at salinities between 150∼250 g/L NaCl, offers distinctive advantages including inherent resistance to contamination, simplified polymer recovery through osmotic cell lysis without the need for organic solvents, and high intracellular PHA accumulation.^16,17^ It should be pointed out that the intrinsic high salinity of crude glycerol aligns with the salt requirements of *H. mediterranei*, potentially reducing or eliminating the need for external salt addition, thus significantly enhancing the economic feasibility of *H. mediterranei*-based PHA production.

Despite these promising advantages, the adoption of *H. mediterranei*-based crude glycerol valorization has been impeded by significant intrinsic inhibition stemming from the complex composition of crude glycerol. Previous studies utilizing *H. mediterranei* for crude glycerol fermentation have required substantial dilution, typically achieving glycerol concentrations of only 10∼20 g/L, to mitigate inhibitory effects sufficiently to enable microbial growth and PHA accumulation.^14,15^ It should be realized that the biodiesel wastewater typically contains glycerol concentrations as high as 300∼600 g/L, which means a minimum of 30× dilution is usually required.^10,14^ Such a high dilution requirement negates the economic benefits derived from the concentrated carbon and high salinity contents of crude glycerol. Although microbial inhibition has generally been attributed to the complex mixtures of impurities in crude glycerol, the specific chemical identities, underlying inhibitory mechanisms, and effective mitigation strategies remain largely unexplored.^18^

In order to fill these critical knowledge gaps, this study hypothesized that chemical pretreatment methods commonly applied in industrial wastewater treatment, particularly overliming and acid precipitation, could mitigate the microbial inhibition associated with high-salinity crude glycerol. Moreover, since arrested anaerobic digestion (aAD) has been commonly used as a pretreatment method to turn heterogeneous feedstocks into readily assimilable substrates such as volatile fatty acids (VFAs) for *H. mediterranei* utilization and in turn PHA production,^19^ it is also hypothesized that the aAD pretreatment method may help reduce the inhibitory compounds. To test these hypotheses, it was proposed that advanced analytical techniques, including liquid chromatography–mass spectrometry (LC-MS), attenuated total reflectance Fourier transform infrared spectroscopy (ATR-FTIR), and ultraviolet–visible spectroscopy (UV–vis), could be harnessed for effectively identifying inhibitory compounds and providing mechanistic insights into their occurrence within biodiesel wastewater. Ultimately, by clarifying the chemical nature of inhibitors and identifying practical mitigation strategies, this research aims to contribute essential knowledge toward scalable and sustainable valorization of crude glycerol into high-value bioplastics using halophilic biotechnology.

## 2 Materials and Methods

### 2.1 Feedstock Preparation

The high-salinity crude glycerol used in this study was derived from settled glycerol sludge obtained from plant-based biodiesel production provided by an industry partner in the United States (USA). The original biodiesel wastewater had a sodium concentration of 85.6 g/L, corresponding to an equivalent salinity of approximately 214 g/L sodium chloride (NaCl). It exhibited average total solids (TS) and volatile solids (VS) contents of 43.3 ± 0.60% and 20.7 ± 0.46%, respectively, with a measured soluble total organic carbon (TOC) concentration of 120 ± 5 g/L and an alkaline pH of approximately 9, as determined by methods described in Section 2.4.3. Prior to microbial fermentation, the wastewater was filtered using 0.22 μm membrane filters and the collected filtrate was stored under 4 °C refrigeration until further use.

The aAD treatment of the high-salinity crude glycerol was performed in semicontinuous, bench-scale anaerobic reactors with total and working volumes of 0.5 and 0.4 L, respectively. These reactors were operated at a hydraulic retention time (HRT) of 12 days for approximately five retention cycles (totaling 62 days). Reactors were inoculated using a 1:1 (v/v) mixture of effluents sourced from semicontinuous, lab-scale (37.8 L) aAD reactors maintained at the Bioenergy & Biotechnology Laboratory (College Park, Maryland, USA).^20^ During the operation, the reactors were fed with fresh high-salinity crude glycerol twice weekly. If reactor pH values dropped below 7.0, adjustments were made weekly by using an 8.5% potassium hydroxide solution to maintain pH neutrality. The organic loading rate (OLR) was maintained at approximately 17 g of VS/L·day. After the final retention cycle, the digestate was collected and passed through 0.22 μm membrane filters, and the resulting filtrate was preserved under refrigerated conditions for subsequent halophilic microbial fermentation experiments.

### 2.2 Dilution and Growth Studies

*H. mediterranei*, an extremely halophilic archaeon (ATCC 33500, American Type Culture Collection, Manassas, VA, USA), was used in this study. To evaluate microbial growth and PHA production, a series of dilution experiments was conducted using either untreated high-salinity crude glycerol or aAD-treated crude glycerol as the carbon and salinity sources. Wastewaters were diluted at five different factors: 3×, 6×, 11×, 23×, and 45×, using deionized water as the diluent. Each diluted feedstock was adjusted to an optimal salinity of approximately 156 g/L NaCl by supplementing additional NaCl on the top of the existing salinity that came with the crude glycerol, suitable for *H. mediterranei* growth. It should be noted that lower dilution factors enabled greater utilization of feedstock-containing salts (approximately 214 g/L NaCl in undiluted crude glycerol), thereby reducing the need for externally supplemented NaCl. For instance, a 3× dilution of crude glycerol containing 71 g/L NaCl provided approximately 46% of the total required salt concentration, necessitating only 54% external supplementation, whereas a 45× dilution containing 5 g/L NaCl provided only about 3%, requiring 97% additional NaCl supplementation. Prior to inoculation, the pH was adjusted to 7.0∼7.2 using 1 M hydrochloric acid or sodium hydroxide. Macro- and micronutrients were supplemented according to ATCC 1176 medium recommendations.^21^ Subsequently, cultures were inoculated at a ratio of 0.2% (v/v) with an actively growing *H. mediterranei* culture, activated according to previously described methods.^22^ All microbial cultivation experiments were performed in triplicate in sterile 500 mL shake-flasks containing 100 mL of medium and incubated aerobically at 37 °C with constant shaking at 150 rpm until the stationary optical density (OD) phase was reached. Microbial growth was monitored using OD at 600 nm (OD_600 nm_) immediately after sampling, and the collected biomass was analyzed for dry cell weight (DCW) and intracellular PHA content.

### 2.3 Inhibition Mitigation Strategies

Overliming, a commonly used chemical pretreatment method to mitigate inhibitory compounds, was evaluated by adjusting the aAD-treated crude glycerol pH to approximately 10∼11 using calcium hydroxide. Following pH adjustment, the solution was incubated at 60 °C for approximately 5∼6 h, with manual shaking conducted hourly to enhance coagulation.^23^ After incubation, the mixture was filtered through 0.22 μm membrane filters to collect the clarified supernatant. This clarified digestate was then readjusted to the optimal pH range of 7.0∼7.2, diluted at 3×, and evaluated for microbial growth and PHA production. Control experiments without the overliming treatment were conducted simultaneously.

Acid precipitation treatment (pH 2 treatment) was conducted by adjusting the pH of both untreated high-salinity crude glycerol and aAD-treated crude glycerol to 2.0 using concentrated sulfuric acid. Following this pH 2 treatment, the samples were shaken at 150 rpm overnight to facilitate complete precipitation. Subsequently, the resulting precipitates floating on top of the solution were separated by filtration through a 0.22 μm membrane filter. The clarified supernatants were then neutralized to a pH range of 7.0∼7.2 using 1 M sodium hydroxide and diluted at either 3× or 6× for microbial growth and PHA production. The precipitates were collected and stored in a freezer for further identification and characterization.

### 2.4 Analytical Techniques

#### 2.4.1 Identification and Characterization of Precipitates

To identify and characterize inhibitory precipitates, a series of analytical approaches employing LC-MS, ATR-FTIR, and UV–vis was applied. Precipitates obtained from the aforementioned pH 2 treatment were initially dissolved in methanol containing 0.1% formic acid. After that, samples were centrifuged at 13,000 × *g* for 10 min to pellet insoluble materials, and the clarified supernatants were analyzed by LC-MS (Model 8060, Shimadzu Corporation, Tokyo, Japan). Chromatographic separation was achieved using a Shimadzu column (3 × 50 mm, 5 μm particle size) at a flow rate of 0.4 mL/min, with gradient elution consisting of water (solvent A, 0.1% formic acid) and acetonitrile (solvent B, 0.1% formic acid). ATR-FTIR spectroscopy (INVENIO, Bruker Corporation, Billerica, MA, USA) was employed to identify the characteristic functional groups present in precipitated inhibitors. Approximately 2 mg of dried precipitate was placed directly onto the ATR diamond crystal, and spectra were recorded over the wavelength range of 400∼4000 cm^–1^ at a resolution of 1 cm^–1^. UV–vis spectroscopy (UV-2550, Shimadzu Corporation, Tokyo, Japan) was performed to qualitatively evaluate compound removal resulting from pH 2 treatment induced precipitation. Filtered liquid samples, collected before and after the removal of precipitates by pH 2 treatment, were diluted 20× and analyzed by UV–vis spectroscopy over a wavelength range of 250∼700 nm.

#### 2.4.2 Quantification of Precipitates

Precipitate concentrations from untreated high-salinity crude glycerol and aAD-treated crude glycerol were comparatively quantified. Precipitates obtained from pH 2 treatment were freeze-dried to a constant weight, and their dry mass was determined gravimetrically. All measurements were performed in triplicate to ensure accuracy and reproducibility.

#### 2.4.3 Characterization of High-Salinity Crude Glycerol

The TS and VS were measured according to the American Public Health Association (APHA) Standard Methods 2540B and 2540E, respectively.^24^ Briefly, TS analysis was conducted by transferring 10 mL of liquid sample into predried porcelain crucibles and drying at 105 °C to constant weight. For VS determination, dried TS samples were ignited in a muffle furnace at 550 °C until a constant weight was obtained. Sodium concentration was analyzed by AgroLab Inc. (Harrington, DE, USA) using the Association of Official Analytical Chemists (AOAC) Official Method 6010B.^25^ TOC was quantified with a TOC analyzer (TOC-LCSN, Shimadzu Corporation, Tokyo, Japan). Samples were prepared by filtering through 0.45 μm syringe filters, followed by dilution with deionized water before analysis.

#### 2.4.4 Determination of Cell Growth and PHA Content

Microbial growth was monitored by measuring OD_600 nm_ using a microplate reader (Synergy H1, BioTek Instruments Inc., Winooski, VT, USA). DCW was measured as the volatile suspended solids (VSS) of the cell broth by following the standard VSS measurement method.^24^ The cell maximum specific growth rate (μ_max_) was estimated from the OD_600 nm_ growth curve using the single interval method.^26^ Intracellular PHA content was quantified using gas chromatography (GC, Model 8890, Agilent Technologies Inc., Santa Clara, CA, USA) following cell disintegration with sodium hypochlorite and methanolysis of dried biomass, according to previously established methods.^22,27^ Briefly, microbial biomass harvested by centrifugation at 8000 × *g* for 10 min was washed sequentially with 4% sodium hypochlorite and deionized water, followed by freeze-drying to constant weight. Approximately 25 mg of dried biomass was subjected to methanolysis by incubation with 2 mL chloroform and 2 mL acidified methanol (3% sulfuric acid) at 105 °C for 120 min. After cooling, 1 mL of deionized water was added to induce phase separation, and the resulting mixture was vortexed and centrifuged at 5000 × *g* for 15 min. The lower chloroform layer was collected, filtered through a 0.22 μm membrane, and analyzed by GC equipped with a flame ionization detector and HP-5 column (30 m × 320 μm × 0.25 μm), using commercial poly­(3-hydroxybutyrate-*co*-3-hydroxyvalerate) (PHBV, Sigma-Aldrich, St. Louis, MO, USA) as standards. The intracellular PHA content (%, w/w) was calculated as the ratio of PHA concentration determined by GC to the DCW.

### 2.5 Statistical Analysis

All experiments were conducted in triplicate, with results reported as the mean ± standard error. Statistical significance between two experimental groups was determined using two-tailed Student’s *t*-tests conducted in Microsoft Excel. Comparisons involving more than two groups were analyzed using ordinary one-way analysis of variance (ANOVA), followed by Tukey’s multiple comparison test in GraphPad Prism (GraphPad Software, San Diego, CA, USA). Differences were considered statistically significant at *p* < 0.05.

## 3 Results

### 3.1 Effect of Dilution Factor and aAD on Crude Glycerol Utilization by *H. mediterranei*

To measure the extent of the inhibition of crude glycerol on *H. mediterranei* growth, a gradient of dilution factors (3×, 6×, 11×, 23×, and 45×) was tested to find the minimum dilution required to mitigate inhibition. Results in Figure 1 indicated negligible *H. mediterranei* growth or PHA synthesis at dilution factors below 23×, confirming significant substrate inhibition from raw crude glycerol. Growth and PHA accumulation commenced from a dilution factor of 23×, establishing this as the minimum dilution experimented in this study that is required to overcome inhibitory effects in the crude glycerol. Increasing dilution further from 23× to 45× resulted in an approximately 50% reduction in biomass production, attributable to low substrate availability under excessively dilute conditions (Figure 1A). For the same reason, the cellular PHA content at the 45× dilution was also lower, comparable to that observed at 23× (Figure 1B). In terms of μ_max_, its values at both 23× and 45× dilution were substantially lower than that of the control group cultivated with ATCC media (Figure S1), indicating there were still inhibitors remaining after such extensive substrate dilution.

**Figure 1:**
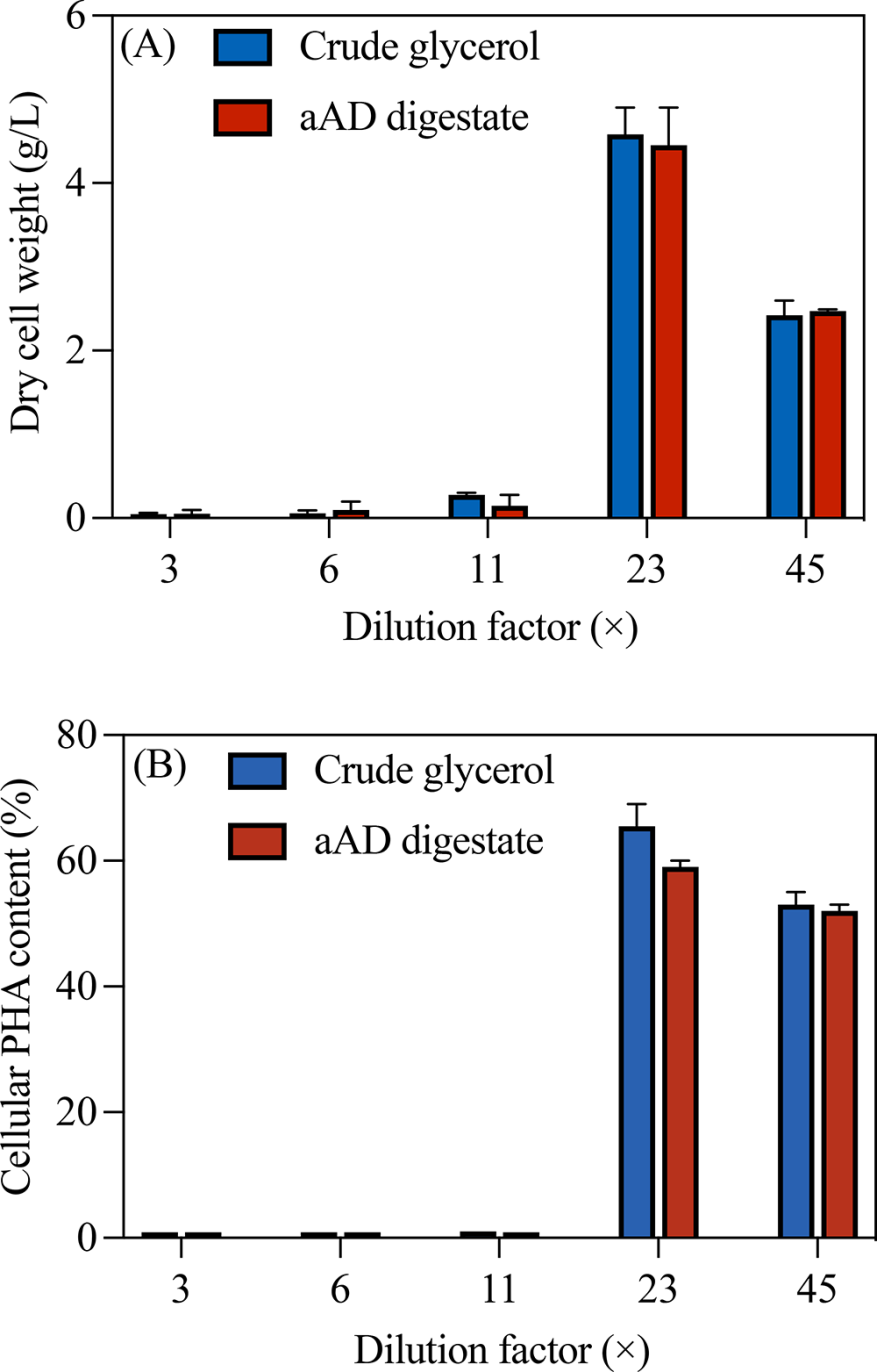
(A) Cell growth and (B) cellular PHA content of *H. mediterranei* cultivated on crude glycerol and aAD-treated crude glycerol (aAD digestate) across a gradient of substrate dilution factors. Note: statistical analysis using *t*-tests showed no significant differences (*p* > 0.05) between the results from crude glycerol and aAD digestate groups at each respective dilution factor.

Parallel trials investigated the efficacy of aAD pretreatment in reducing inhibitory compounds in crude glycerol. Visual inspection in Figure 2 indicated partial color removal by aAD treatment, as aAD-treated crude glycerol in Figure 2B showed lighter color intensity compared to the crude glycerol in Figure 2A. Likewise, the μ_max_ values measured in aAD-treated crude glycerol were consistently higher than those in raw crude glycerol, regardless of the dilution factor, suggesting that the aAD treatment might have removed a certain amount of inhibitors (Figure S1). However, *t*-tests on Figure 1A,B data revealed no significant differences (*p* > 0.05) in terms of final *H. mediterranei* biomass growth or PHA accumulation with and without aAD-treatment at respective dilution factors. These findings demonstrated that aAD pretreatment alone did not effectively mitigate enough inhibitory constituents present in crude glycerol to improve the production of cell and PHA. This can be further inferred from the double cell growth achieved at 23× over that at 45×, which is almost proportional to the dilution factor difference between the two groups (Figure 1). Collectively, these results highlight the necessity of a minimum dilution factor of approximately 23× to permit microbial growth and PHA biosynthesis from crude glycerol (Figure 1). The limited efficacy of aAD indicates a need to explore alternative pretreatment or inhibitor-removal strategies to optimize crude glycerol valorization (Figure 1).

**Figure 2:**
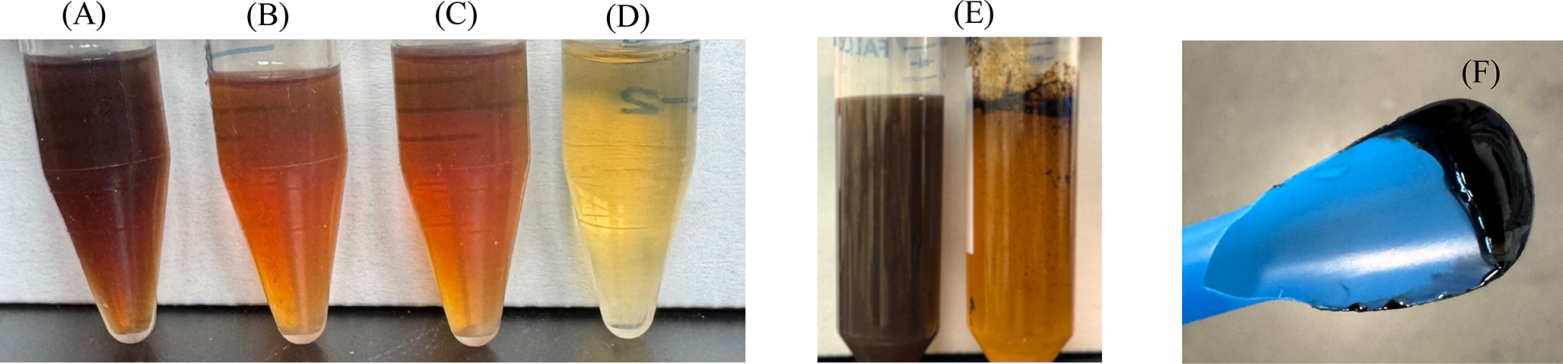
Visual appearance of the supernatant from (A) untreated crude glycerol, (B) aAD-treated crude glycerol, (C) aAD-treated crude glycerol after overliming, and (D) aAD-treated crude glycerol after pH 2 treatment and precipitate removal; (E) comparison of crude glycerol before (left) and after (right) pH 2 treatment, and (F) dark, slimy precipitate formed following pH 2 treatment.

### 3.2 Mitigation Strategy Development

Because inhibitory compounds are usually unknown, to mitigate substrate inhibition, simple strategies such as overliming and acidification have been commonly used in literature for removing inhibitory compounds.^28^ Therefore, both methods were tested on aAD-treated crude glycerol. Visual inspection revealed that overliming treatment did not result in notable changes to the aAD-treated crude glycerol (Figure 2B,C), while pH 2 treatment induced the formation of dark, slimy precipitates that floated on the top of the treated solution similar to the observation in Figure 2E. The filtration of this pH 2 treated glycerol solution allowed harvesting of this oily dark matter on spatula as shown in Figure 2F. The filtrate collected in Figure 2D showed the lightest color among all treated samples, indicating the darkest matter removal. This dark precipitate formed in aAD-treated crude glycerol was quantified at TS around 3.68 ± 0.11 g/L. Subsequently, *H. mediterranei* growth and PHA accumulation were assessed in aAD-treated crude glycerol diluted by a factor of 3×, with and without overliming or pH 2 treatment (Figure 3A). Results indicated negligible *H. mediterranei* growth in both untreated and overliming-treated groups (*p* > 0.05), confirming that overliming had limited impact on inhibitor removal as shown in Figure 3A. Thus, overliming was not included in subsequent experiments. In contrast, pH 2 treatment allowed *H. mediterranei* to grow in 3× diluted aAD-treated crude glycerol. However, the 3× diluted aAD-treated crude glycerol without pH 2 treatment in the control did not allow any *H. mediterranei* growth. The differences in terms of DCW and PHA content observed in the pH 2 treatment group were statistically significant compared to the overliming group (*p* < 0.05) and no treatment group (*p* < 0.05), suggesting that the slimy precipitate formed after pH 2 treatment as shown in Figure 2 might be a kind of inhibitory compounds that have been removed. This corroborates the observation in Figure S1 that, with pH 2 treatment, the μ_max_ measured in 3× diluted aAD-treated crude glycerol was greater than those measured at higher dilutions (23× and 45×) with or without aAD treatment and only 20% lower than the positive control, underscoring the essential role of pH 2 treatment in inhibitor removal.

**Figure 3:**
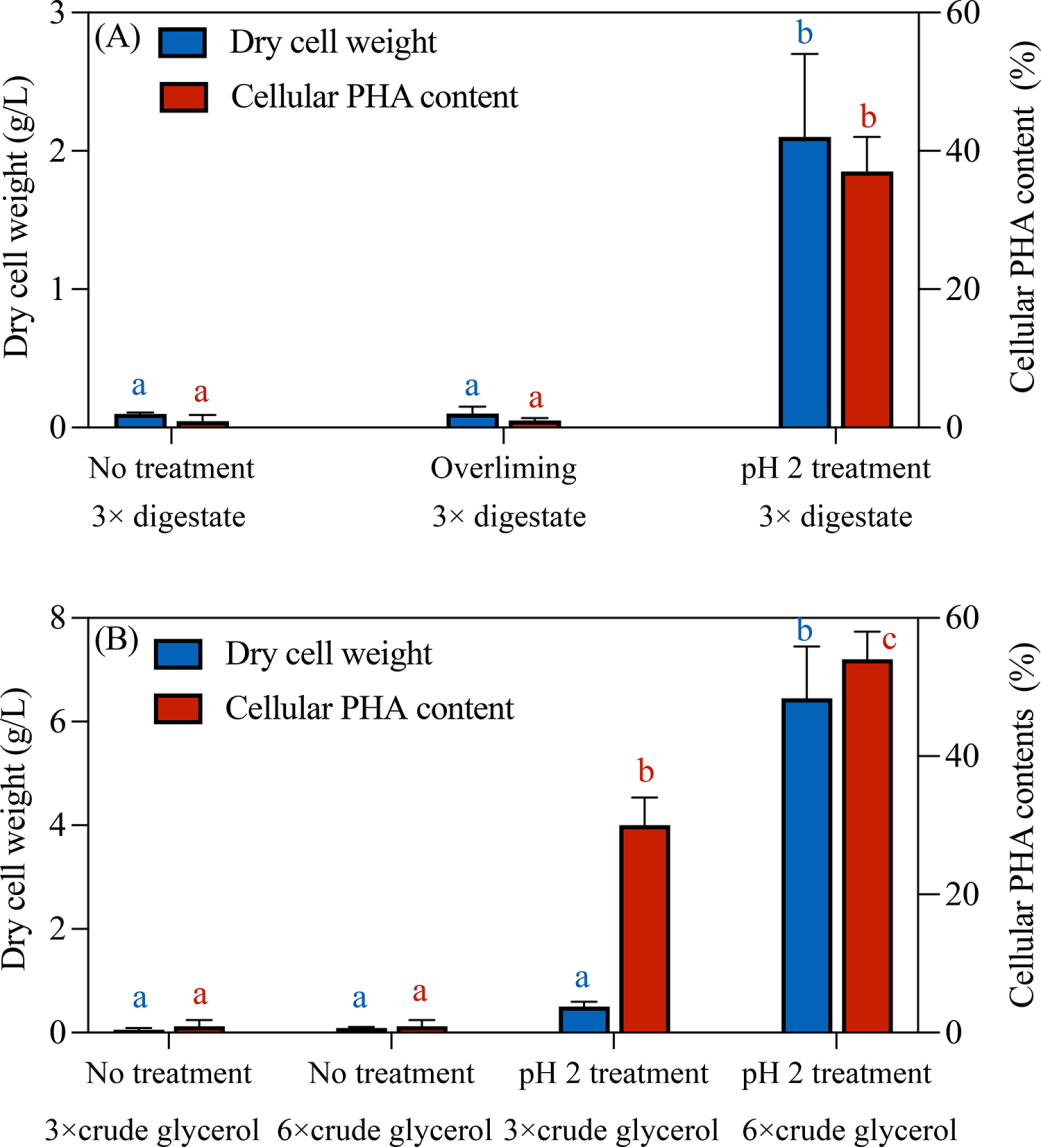
Cell growth and cellular PHA content of *H. mediterranei* cultivated in (A) 3× diluted aAD-treated digestate supernatant without other treatment, with overliming treatment, and with pH 2 treatment; and (B) raw crude glycerol with 3× and 6× dilution with and without pH 2 treatment. Note: the different letter (a, b, c) above each bar indicates statistically significant differences between groups (*p* < 0.05) analyzed by ANOVA followed by Tukey’s multiple comparison test. Blue letters represent DCW results, and red letters represent PHA content results. Groups sharing the same letter and color are not significantly different from each other (*p* > 0.05).

To double confirm that the dark, slimy precipitate removed from the aAD-treated crude glycerol is indeed responsible for the inhibition of *H. mediterranei* growth in Figure 3A, these dark matters shown in Figure 2F were redissolved at pH 11 into the aAD digestate where they were precipitated out of and then fed both aAD digestates with and without the dark matter redissolution to *H. mediterranei*. Results in Figure 4 consistently showed that the aAD-treated crude glycerol with dark precipitate removal was able to allow *H. mediterranei* to grow, as well as the positive control fed with ATCC substrate without substrate inhibition. Specifically, the OD_600 nm_ in the aAD-treated crude glycerol with dark precipitate removal quickly grew from 0 to 4 and then plateaued after 168 h of incubation, which was even greater than the OD_600 nm_ in the positive control. However, after reintroducing the dark precipitate into the same aAD digestate, *H. mediterranei* was not able to grow any more (Figure 4), which is in line with the groups without pH 2 treatment. The negative control group using ATCC substrate without inoculation did not show any growth, proving the satisfactory sterilized condition during the experiment. Therefore, it can be confirmed that the dark, slimy substances removed from the aAD-treated crude glycerol were indeed the inhibitory compounds suppressing *H. mediterranei* growth in Figures 1 and 3.

**Figure 4:**
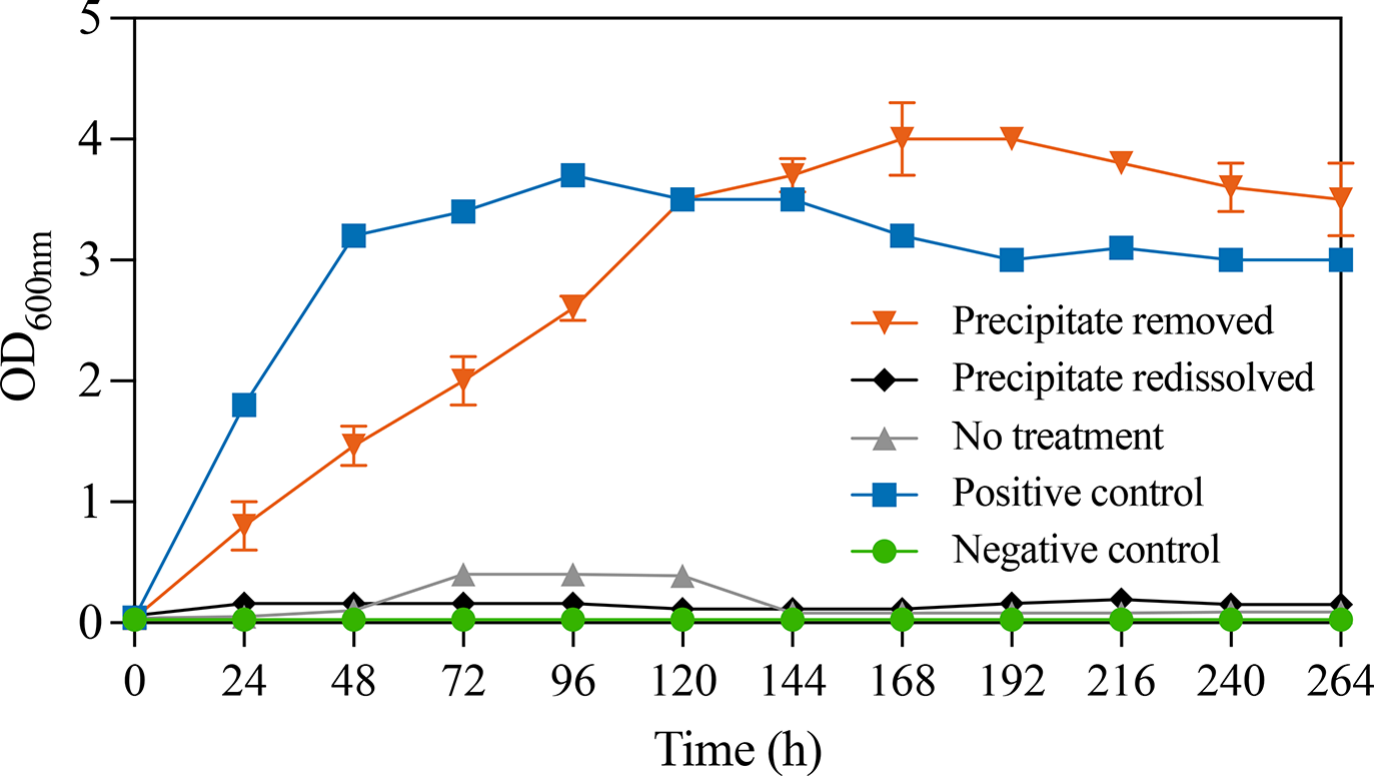
OD profiles of *H. mediterranei* during cultivation on 3× diluted aAD-treated crude glycerol supernatant with precipitate removed, with precipitate redissolved, and without other treatment compared to positive control (ATCC media with inoculation) and negative control (ATCC media without inoculation).

To further confirm whether these inhibitory compounds existed before or after aAD, pH 2 treatment was also performed on raw crude glycerol without aAD. A similar dark, slimy precipitate was also formed in raw crude glycerol after pH 2 treatment (Figure 2E) but at a greater TS quantity of 4.05 ± 0.13 g/L. LC-MS chromatogram (Figure S2) confirmed identical chemical composition of precipitates from the raw and aAD-treated crude glycerol. However, unlike the aAD-treated crude glycerol, raw crude glycerol required slightly greater dilution (6× rather than 3×) to support *H. mediterranei* growth after precipitate removal, as no significant difference in DCW was found between the 3× dilution and no treatment groups (*p* > 0.05) (Figure 3B). Furthermore, the μ_max_ measured in 6× diluted crude glycerol without aAD treatment was only 33% of that measured in 3× diluted crude glycerol with aAD treatment (Figure S1). The less dark precipitates, the lower minimum dilution factor required, and the greater μ_max_ measured with aAD-treated crude glycerol suggested that aAD might have removed the 0.37 g/L inhibitory compounds, which contributed to 9% reduction of inhibitors over the 4.05 ± 0.13 g/L that can be precipitated before aAD.

### 3.3 Inhibitor Identification

Having confirmed the inhibitory nature of the acid-induced precipitate, further chemical characterization was conducted by advanced spectroscopic analyses. First, the effectiveness of inhibitor removal was investigated by using UV–vis spectroscopy (Figure 5A). The UV–vis analysis revealed a notable decrease in absorbance over a broad wavelength range of 250∼500 nm following the precipitate removal. This observation qualitatively confirmed the substantial removal of substances from the digestate solution by pH 2 precipitation.^29^ Second, since the initial solubility assays indicated that the precipitate has a hydrophobic lipid-like composition because the precipitate was fully soluble in methanol but insoluble in neutral pH aqueous solution,^30^ LC-MS employing a lipid-specific analytical column was used for precise molecular identification of the dark precipitate shown in Figure 2F. The resulting LC-MS chromatogram (Figure 5B) revealed distinct peaks with substantially greater intensity than other signals, clearly identifying them as the primary constituents of the inhibitory precipitate. Detailed analysis of mass-to-charge (*m*/*z*) ratios identified the inhibitory compounds to be LCFAs, specifically including an 18-carbon fatty acid containing one carbon–carbon double bond and two additional oxygens (FA 18:1:O2; *m*/*z* 313.2323), an 18-carbon saturated fatty acid with two additional oxygens (FA 18:0:O2; *m*/*z* 315.2476), an 18-carbon fatty acid with one carbon–carbon double bond and one additional oxygen (FA 18:1:O; *m*/*z* 297.2371), and a 19-carbon fatty acid with one carbon–carbon double bond and three additional oxygens (FA 19:1:O3; *m*/*z* 343.2417).^31^ These LCFAs are typically contained in crude glycerol from biodiesel production due to the incomplete transesterification to fatty acids and methyl esters (biodiesel). Third, complementary analysis using ATR-FTIR spectroscopy of the precipitate (Figure 5C) supported the LC-MS findings by confirming key functional groups. Characteristic absorption bands at approximately 2920 and 2850 cm^–1^ corresponded to aliphatic C–H stretching vibrations, typical of fatty acid hydrocarbon chains.^32^ A prominent peak at around 1705 cm^–1^ indicated the presence of carbonyl (C=O) functionalities associated with carboxylic acid groups, while a broad absorption at approximately 3300 cm^–1^ suggested hydroxyl (−OH) groups consistent with fatty acid structures.^33^ In the fingerprint region, a merged CH_2_-scissoring/CH_3_-bending hump at ∼1400 cm^–1^ and a modest C–O stretch at ∼1100 cm^–1^ suggest the presence of minor mono/diglyceride residues.^34^ In summary, these integrated analytical results collectively confirmed the removal of inhibitory compounds as a result of the pH 2 treatment and identify the inhibitory precipitate as predominantly composed of LCFAs (primarily C18 and C19 fatty acids), which also explains why the dark precipitate can float on the surface of the aqueous phase because LCFAs usually have a density lower than that of water (Figure 2E).

**Figure 5:**
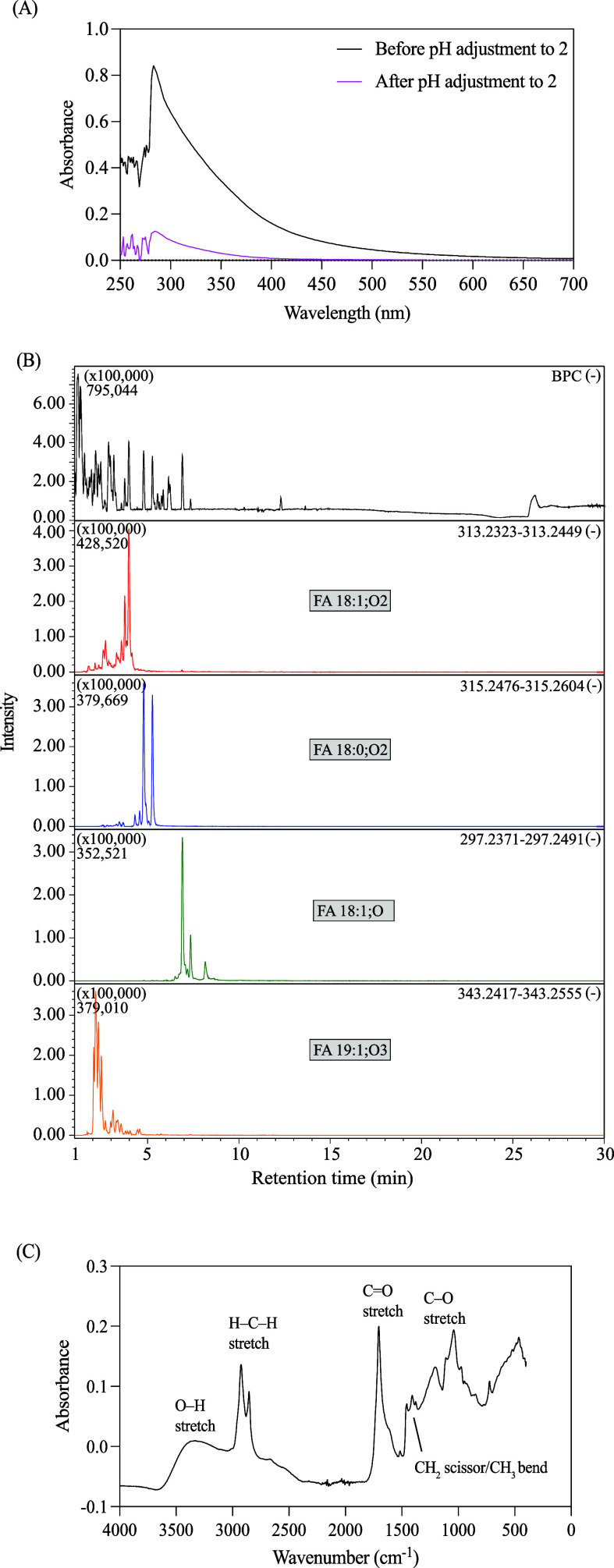
(A) UV–vis absorption spectra of aAD-treated crude glycerol before and after pH 2 treatment; (B) LC-MS chromatogram; and (C) ATR-FTIR spectrum of the dark precipitate formed after pH 2 treatment.

## 4 Discussion

### 4.1 Mechanism of LCFA Formation, Removal, and Inhibition

In view of the processes used during the transesterification reaction for biodiesel production, it is not surprising to observe LCFA contained in the final crude glycerol as shown in Figure 5. There are at least three sources contributing to LCFA occurrence in the course of biodiesel production,^35–37^ i.e., (i) the feedstock oil used for biodiesel production might come with LCFAs, (ii) the stoichiometry of triglyceride hydrolysis under alkaline reaction conditions is also known to produce LCFAs, and (iii) the saponification during biodiesel production generates the LCFA salts that can be protonated into LCFAs at low pH. As observed experimentally (Figures 2–5), pH 2 treatment causes the protonation of these ionized, water-soluble fatty acids, rendering them hydrophobic and insoluble, leading to their precipitation. The resulting precipitate exhibits pronounced viscosity and hydrophobic characteristics, presenting visually as dark, slimy aggregates due to impurities adsorbed from crude glycerol (Figure 2F).

The inhibitory effect of LCFAs on microbial systems is well-documented and primarily attributed to their hydrophobic nature, which enables them to adsorb onto microbial cell membranes, disrupt the membranes’ structure and function, and thereby impair essential cellular functions such as nutrient uptake and metabolic processes.^38,39^ Although AD is known to be able to break down LCFAs into VFAs, a prolonged digestion time, e.g., 25∼30 days, is usually required because LCFAs are relatively more degradation resistant to microbial hydrolysis than their carbohydrate and protein counterparts in organic matters.^40^ For this reason, it is not difficult to understand that the aAD with only 12-day HRT was able to degrade only 9% LCFA as described in previous sections. Moreover, the high salinity property added even more challenges to the biodegradability of those LCFA-rich wastewaters, which requires alternative methods for improving its utilization by *H. mediterranei*. The fact that pH 2 treatment can remove these inhibitory compounds, reduce the minimum dilution factor of crude glycerol required for *H. mediterranei* growth from 23× to 3×, and increase μ_max_ from 0.08 to 0.12 suggests that pH 2 treatment could be a preferred strategy for improving the crude glycerol conversion to PHA via *H. mediterranei* metabolism (Figures 1, 3, and S1).

### 4.2 Implication of the Potential of Full-Scale Crude Glycerol Valorization by *H. mediterranei*

Although *H. mediterranei* is known to be able to directly utilize crude glycerol as carbon source for PHA production,^14,41^ the applicability of this approach has been limited by the high dilution factors that have to be used to mitigate the intrinsic inhibitors that came with the crude glycerol. This study for the first time demonstrated that pH 2 treatment effectively reduced inhibitory compounds, enabling a substantial reduction in the required dilution factor from approximately 23× to 3× with aAD or to 6× without aAD (Figures 1–4). A direct positive outcome from this dilution factor reduction is the PHA titer increase of 40%, as derived by multiplying the DCW and cellular PHA content presented in Figures 1 and 3. The improved titer can be attributed to greater concentrations of carbon substrates made available to *H. mediterranei* as a direct consequence of the reduced dilution. On the top of this benefit, substantial salt savings can be achieved too when leveraging the high salinity that comes with crude glycerol. Given that optimal PHA biosynthesis in *H. mediterranei* typically requires salinities of around 150∼250 g/L NaCl,^17^ supplying and subsequently managing such high-salinity media contributes substantially to the minimum selling price of PHAs.^42^ Thus, chemical or energy-intensive treatment methods have been explored to allow salt recycling for *H. mediterranei* fermentation.^43,44^ In this context, the present study’s finding revealed that a portion of the indigenous salt (214 g/L NaCl) contained in crude glycerol can be taken advantage of for the salinity provision in *H. mediterranei* fermentation. Given the dilution factor reduction from approximately 23× to 3×, an estimate of 46% salt supply can be offset by the indigenous salt in crude glycerol, which directly translates to corresponding reductions in salt procurement and salty wastewater treatment costs. Consequently, pH 2 treatment emerges as a highly promising pretreatment strategy, not only mitigating inhibitors but also enhancing overall economic feasibility and environmental sustainability.

Nonetheless, the current pH 2 treatment strategy did not eliminate all inhibitory components, as residual inhibition still necessitates at least a 3× dilution, at which μ_max_ remained lower than that of the control group cultivated in ATCC media (Figures 3 and S1). This is still a limiting factor for further PHA titer improvement. Besides, the small flask volume (100 mL working volume) used in this study did not allow PHA productivity to be meaningfully measured. Going forward, efforts should be made in pilot-scale studies to identify and remove other potential inhibitors that cannot be removed by the pH 2 treatment method used in this study and evaluate the performance improvement toward both PHA titer and productivity. Certainly, the fed-batch strategy also can be considered to further mitigate the inhibitory effect for titer enhancement due to its automatic substrate dilution. The fact that aAD was able to biologically remove some of the inhibitors indicates potential for a fed-batch cultivation strategy, because slowly bleeding crude glycerol into a fed-batch reactor may allow real-time inhibitor removal before they are accumulated to the extent that is high enough for complete inhibition of *H. mediterranei* growth.

## 5 Conclusions

This study provides critical insights into the sustainable valorization of high-salinity crude glycerol generated from biodiesel production into PHAs by the halophilic archaeon *H. mediterranei*. For the first time, LCFAs were conclusively identified as the principal inhibitors that suppress microbial growth and PHA biosynthesis from crude glycerol. aAD, tested as a biological pretreatment method, showed limited effectiveness, removing only approximately 9% of the LCFA inhibitors. Furthermore, chemical pretreatment via overliming did not demonstrate a substantial capability for inhibitor mitigation. In contrast, pH 2 treatment substantially reduced microbial inhibition and lowered the required dilution factor from 23× to 3×. This dilution reduction directly enhanced PHA titers for 40% and improved salt saving for 46%, substantially decreasing salt input costs for PHA production from *H. mediterranei*. Future work is warranted to further identify and remove other types of inhibitors that are still contained in high salinity crude glycerol and, in turn, evaluate their impacts on PHA productivity and titer at the pilot scale for further PHA production enhancement along with more salt savings.

## Supplementary Material



## Notes

Maximum specific growth rate (μ_max_) data of *Haloferax mediterranei* cultivated on raw and arrested anaerobic digestion (aAD)-treated crude glycerol under different pretreatment and dilution conditions; liquid chromatography–mass spectrometry (LC-MS) chromatograms of precipitates formed after pH 2 treatment (PDF)
