# Supplementary material for: Identification and Mitigation of Inhibitory Substances Contained in High-Salinity Crude Glycerol Generated from Biodiesel Production for Polyhydroxyalkanoate Synthesis by Haloferax mediterranei
Source: ACS Sustain Chem Eng. 2025 Sep 22;13(39):16403–12. doi: 10.1021/acssuschemeng.5c05475 (PMC12505262; doi:10.1021/acssuschemeng.5c05475)
Supplement: Supplementary file 1 [file sc5c05475_si_001.pdf]

Supporting information

Identification and Mitigation of Inhibitory Substances Contained in High-Salinity Crude Glycerol Generated from Biodiesel Production for Polyhydroxyalkanoate Synthesis by *Haloferax mediterranei*

Xueyao Zhang[a], Richard F. Helm[b], Emily L. McCoy[c], Fujunzhu Zhao[d], Mingxi Wang[a], Yebo Li[e], Stephanie Lansing[c], Haibo Huang[d], Zhiwu Wang[a,*]

[a] Department of Biological System Engineering, Virginia Polytechnic Institute and State University, Blacksburg, VA, 24061, USA
[b] Department of Biochemistry, Virginia Polytechnic Institute and State University, Blacksburg, VA, 24061, USA
[c] Department of Environmental Science and Technology, University of Maryland, College Park, MD, 20742, USA
[d] Department of Food Science & Technology, Virginia Polytechnic Institute and State University, Blacksburg, VA, 24061, USA
[e] Quasar Energy Group, Wooster, OH, 44691, USA

[*]Corresponding author: Zhi-Wu Wang
Address: Human & Agricultural Biosciences Bldg.1, Room 302A, 1230 Washington St. SW, Blacksburg, VA 24061, USA
Email: wzw@vt.edu
Phone: 571-271-1757

The Supplementary Information (SI) includes maximum specific growth rate (μ_{max}) data of *Haloferax mediterranei* cultivated on raw and arrested anaerobic digestion (aAD)-treated crude glycerol under different pretreatments and dilutions, and liquid chromatography-mass spectrometry (LC-MS) chromatograms of precipitates formed after pH 2 treatment.

The supporting information contains the following details:

Number of Pages: 3

Number of Figures: 2

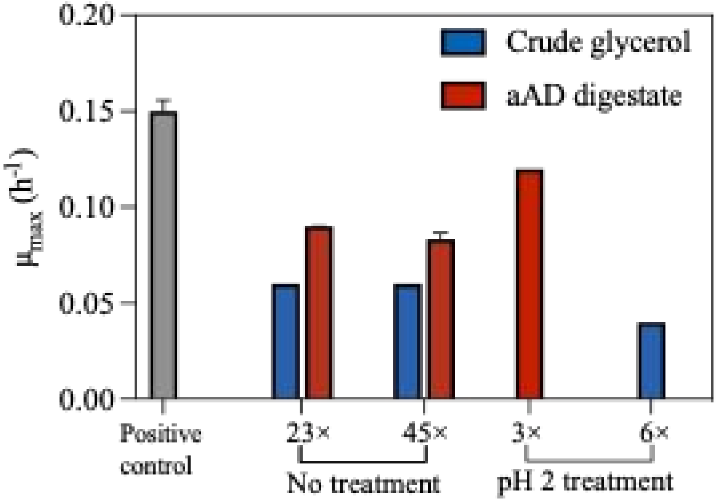

Figure S1. Maximum specific growth rate (μ_{max}) of *H. mediterranei* cultivated on crude glycerol and aAD-treated crude glycerol (aAD digestate) under different dilution factors and pretreatment conditions. The positive control is the ATCC media with inoculation.

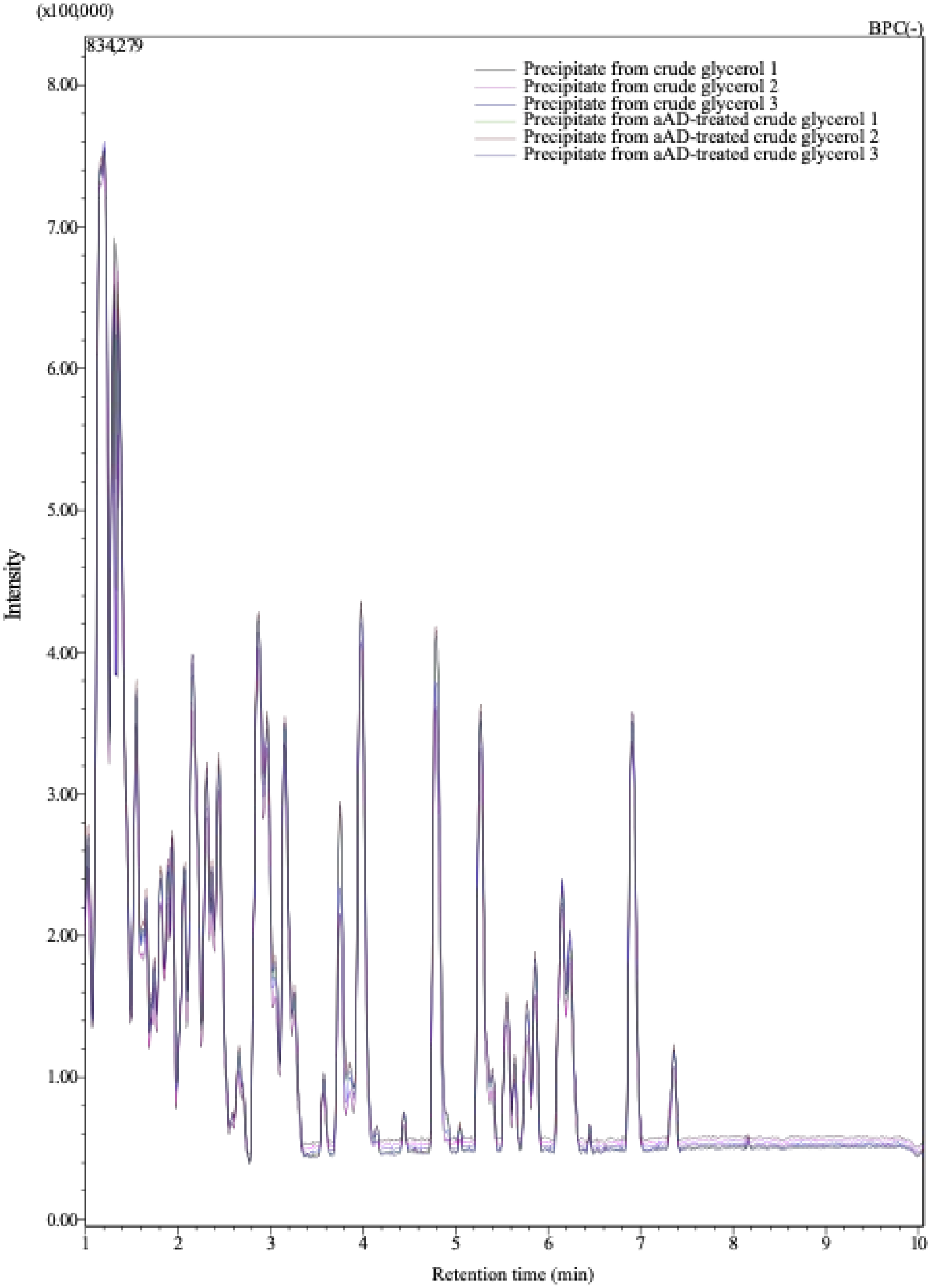

Figure S2. Triplicate comparison of LC-MS chromatogram of the precipitates formed from crude glycerol and aAD-treated crude glycerol after pH 2 treatment.
